# The Prevalence and Risk Factors for Depression Symptoms in a Rural Chinese Sample Population

**DOI:** 10.1371/journal.pone.0099692

**Published:** 2014-06-11

**Authors:** Xinghu Zhou, Bo Bi, Liqiang Zheng, Zhao Li, Hongmei Yang, Hongjie Song, Yingxian Sun

**Affiliations:** 1 Department of Cardiology, The First Hospital of China Medical University, Shenyang city, Liaoning province, China; 2 Department of Psychology, The First Hospital of China Medical University, Shenyang city, Liaoning province, China; 3 Department of Clinical Epidemiology, Shenjing Hospital of China Medical University, Shenyang city, Liaoning province, China; Old Dominion University, United States of America

## Abstract

**Background:**

It is essential to understand how we can prevent and treat the epidemic of depression. Several studies have reported the prevalence of depressive symptoms in the urban population in China, but there is a lack of information regarding the prevalence of depression in rural populations.

**Objective:**

To understand the prevalence of depression in a rural Chinese population and to analyze the risk factors for depression.

**Methods:**

This study used a cross-sectional approach. A total of 11,473 subjects were surveyed and completed the Patient Health Questionnaire-9 (PHQ-9) and the World Health Organization Quality of Life Brief scales. Living conditions, per capita income, marital status, and information about dietary health and chronic disease status were assessed.

**Results:**

The prevalence of depressive symptoms in the population was 5.9%. The prevalence in women (8.1%) was higher compared with men (3.5%) and also increased with age. The per capita income level, amount of sleep obtained per day, education level, weekly consumption of meat and beans or bean products, salt intake, and chronic disease status were associated with depressive symptoms. The quality of life of individuals with a score less than 10 points on the PHQ-9 was significantly better compared with individuals with a score greater than 10.

**Conclusion:**

The prevalence of depressive symptoms among rural population is higher than some southern cities in China. Dietary patterns may be an important risk factor linked to this disorder in the Chinese rural population.

## Introduction

Depression is a world-wide public health issue, and its prevalence increases each year. It is essential to understand depression in order to discover ways to prevent and treat it. Epidemiological studies of mental disorders in China were initiated later compared with western countries. Over the past few decades, several large-scale epidemiological studies have been conducted under the leadership of the government, and there have also been regional epidemiological investigations. However, because of the different diagnostic and screening tools and criteria, epidemiological data are significantly affected by regional factors; thus, the results across studies are different. Although several epidemiological studies on mental disorders have been conducted, there are large differences in the prevalence of depression across different countries and regions [1]–[4].

Many epidemiological studies have shown that the prevalence of depression in females is two times higher compared with males [5], [6]. Most studies that examined the prevalence of depression in China in recent years have focused on populations in cities of China or rural elderly individuals [7]–[12]. Few studies have examined the middle-aged population. Further, more than half of the population lives in rural areas in China, poverty, engages in heavy physical labor, left behind and inadequate investment in health care were common feature of this group. More epidemiological investigations in the rural communities are needed, which would provide reliable information for depression prevention and control in China. The purpose of this study was to investigate the prevalence of depressive symptoms in individuals over 35 years of age in country-side areas in China and to analyze the influencing factors of depressive symptoms in order to verify whether the risk factors for depression that have been identified in other studies are also applicable to China.

## Methods

### Study Population

Liaoning Province is located in northeast China. From July 2012 to August 2013, a representative sample aged ≥35 years was selected to determine the prevalence, incidence and natural history of cardiovascular risk factors in rural areas of Liaoning Province. The study adopted a multi-stage, randomly stratified cluster-sampling scheme. In the first stage, 3 counties (Dawa, Zhangwu, and Liaoyang County) were selected from the eastern, southern, and northern regions of Liaoning province. In the second stage, one township was randomly selected from each county (a total of 3 townships). In the third stage, 8–10 rural villages from each township were randomly selected (a total of 26 rural villages). All eligible permanent residents aged ≥35 years from each village were invited to participate in the study (a total of 14,016 participants). Of this sample, 11,956 participants agreed to participate and completed the study (response rate: 85.3%). The study was approved by the Ethics Committee of China Medical University (Shenyang, China). All procedures were performed in accordance with ethical standards. Written consent was obtained from all participants after they had been informed of the objectives, benefits, medical items and confidentiality agreement regarding personal information. If the participants were illiterate, we obtained the written informed consents from their proxies. The study was completed with the support of the local government and the health administrative departments.

### Data Collection and Measurements

Data were collected during a single clinic visit by a doctor and trained nurses using a standard questionnaire administered via face-to-face interview. Before the survey was administered, all eligible investigators attended a training that included the purpose of this study, how to administer the questionnaire, the standard method of measurement, the importance of standardization, and the study procedures. A test was administered after the training, and only individuals who scored perfectly on the test were eligible to become investigators. During data collection, the investigators received further instructions and support.

Data on the demographic characteristics, lifestyle risk factors, dietary habits, family income, family history of cardiovascular disease, medical history of hypertension, evaluation of psychological status (Patient Health Questionnaire-9; PHQ-9), and quality of life (World Health Organization Quality of Life Brief; WHOQOL-BREF) were obtained by an interview with a standardized questionnaire. There was a central steering committee with a subcommittee for quality control.

### Measuring Depression Symptoms

Depression symptoms were assessed with the PHQ-9, which is widely used in primary health centers for the screening of depression [13], [14]. Each of the nine PHQ depression items corresponds to one of the DSM-IV diagnostic criterion for symptoms for major depressive disorder [15]. The subjects were asked how often, over the past 2 weeks, they had been bothered by each of the depressive symptoms. The response options were “not at all”, “several days”, “more than half the days”, and “nearly every day” and were scored as 0, 1, 2, and 3, respectively. PHQ-9 scores range from 0 to 27, with scores of ≥5, ≥10, and ≥15, representing mild, moderate, and severe levels of depression severity [16]. The psychometric properties of the PHQ-9 are well documented [17]. A cut-off score of 10 is utilized, which has a high level of heterogeneity. Individuals with a PHQ-9 score greater than 10 are considered to be suffering from severe depression symptoms [18].

### Quality of Life

To assess the quality of life of the survey respondents over the previous 4 weeks, there were 27 questions with the addition of one national question during the Turkish reliability study. The first question assessed the perceived quality of life, and the second question assessed the perceived health status. The responses were scored between 0–5. The four domain scores were calculated using the questions subsequent to the first two. The content of the five domains used in the scale included the Physical Health Domain (7 items), the Psychological Health Domain (4 items), the Social Relations Domain (3 items) and the Environmental Domain (6 items). Quality of life increases as the scores rise. According to the WHOQOL-BREF instruction manual, the score of each dimension should be added and converted into a score of 3 to 40; the higher the score, the better the description of the functional status of the dimension, and the higher the quality of life.

### Definitions

The participants reported their occupational physical activity according to the following three categories: (1) light = physically very easy, e.g., sitting office work, such as a secretary; (2) moderate = work that includes standing and walking, e.g., store assistant; and (3) active = work that includes walking and lifting or heavy manual labor, e.g., industrial work, farm work. The dietary pattern was assessed using the recall of foods eaten in the previous year. The questionnaire included questions on the average consumption of several food items per week. The reported consumption was quantified approximately in terms of grams per week (vegetable consumption: rarely = 3, <1,000 g = 2, 1,000–2,000 g = 1, and ≥2,000 g = 0; meat consumption including red meat, fish, and poultry: rarely = 0, <250 g = 1, 250–500 g = 2, and ≥500 g = 3; fried food intake: <1 times = 0, 2–3 times = 1, and ≧4 times = 2; beans or bean products: rarely = 0, 2–3 times = 1, and ≧4 times = 2).

### Statistical Analysis

The present study was designed to provide accurate estimates of the prevalence of depression symptoms according to age and sex in the general Chinese rural population of individuals 35 years or older. A total of 11,956 individuals completed all of the assessments, including the 483 individuals for whom missing data were excluded. Finally, 11,473 cases were used in the data analysis.

The X^2^ test was used to measure associations of each study variable. The prevalence and 95%CIs of the depression symptoms by various criteria were estimated by subgroup and the overall population. The mean values for lifestyle and metabolic factors were determined according to sex and plasma glucose categories. The prevalence estimates for depression symptoms for the overall population and different genders were calculated according to age and other variables. Univariate and multivariate logistic regression analyses were used to identify the independent factors of depression with odds ratios (ORs), and the corresponding 95% CIs were calculated. All statistical analyses were performed using SPSS version 19.0 software, and P values less than 0.05 were considered to be statistically significant.

## Results

### Demographic Characteristics of the Study Subjects

A total of 11,956 subjects were investigated in this study, of which, 11,473 subjects completed the entire study. The mean age of the study subjects was 53.72 years, and the sample population included 5,318 men (mean age: 54.26) and 6,155 women (mean age: 53.26). The demographic characteristics of the three groups are shown in Table 1.

**Table 1:** General Characteristics of the Chinese Rural Population.

	Total, n(%)	Men, n(%)	Women, n(%)	P value
Age group				
35–44	274(23.9)	1216(22.9)	1529(24.8)	<0.001
45–54	3607(31.4)	1624(30.5)	1983(32.2)	
55–64	3424(29.8)	1615(30.4)	1809(29.4)	
≥65	1697(14.8)	863(16.2)	834(13.5)	
Smoking status				
Yes	4044(35.2)	3030(57.0)	1014(16.5)	<0.001
No	7429(64.8)	2288(43.0)	5141(83.5)	
Drinking status				
Yes	2588(22.6)	2408(45.3)	180(2.9)	<0.001
No	8885(77.4)	2910(54.7)	5975(97.1)	
Marital Status				
Married	10606(92.4)	4949(93.1)	5657(91.9)	<0.001
Single	192(1.7)	141(2.7)	51(0.8)	
Widowed	675(5.9)	228(4.3)	447(7.3)	
Education level				
≤Primary school	5662(49.4)	2192(41.2)	3470(56.4)	<0.001
Middle school	4722(41.2)	2513(47.3)	2209(35.9)	
>High school	1089(9.5)	613(11.5)	476(7.7)	
Occupational physical activity				
Light	4163(36.3)	1562(29.4)	2601(42.3)	<0.001
Moderate	2207(19.2)	1010(19.0)	1197(19.4)	
Severe	5103(44.5)	2746(51.6)	2357(38.3)	
Annual income				
≤5000	1432(12.5)	716(13.5)	716(11.6)	0.012
5000–20000	6249(54.5)	2859(53.8)	3390(55.1)	
>20000	3792(33.1)	1743(32.8)	2049(33.3)	
Chronic diseases*				
Yes	2780(24.2)	4225(79.4)	4468(72.6)	<0.001
No	8693(75.8)	1093(20.6)	1687(27.4)	

*Chronic diseases include heart disease, stroke, kidney disease and diabetes.

P value was calculated by the chi-square.

### Prevalence of Depressive Symptoms

The prevalence rates of depressive symptoms at different levels according to the gender and age group are shown in Table 2. The prevalence of depressive symptoms in the entire population was 5.9%, and the depressive symptoms were higher in women (8.1%) compared with men (3.4%). The prevalence of depressive symptoms increased with age for both sexes and decreased with annual income and education level. The prevalence of depression symptoms was at the lowest value among people who slept 7–8 hours per day. Further, the lowest prevalence rates was also found when vegetable intake was at the level of 1,000–2,000 g per week, meat intake was 250–500 g per week, fried food intake was 2–3 times per week, and bean or bean product intake was 2–3 times per week.

**Table 2:** Estimated Prevalence of Depression Symptoms in Chinese Adults.

	Male			Female			Total		
	N	Pre(%)	95%CI	N	Pre(%)	95%CI	N	Pre(%)	95%CI
Overall	5318	3.4	2.9–3.9	6155	8.1	7.4–8.8	11473	5.9	5.5–6.3
Age group									
35–44	1216	2.4	1.5–3.3	1529	4.0	3.0–5.0	2745	3.3	2.6–4.0
45–54	1624	2.8	2.0–3.6	1983	7.4	6.2–8.6	3607	5.3	4.6–6.0
55–64	1615	4.2	3.2–5.2	1809	9.7	8.3–11.1	3424	7.1	6.2–8.0
≥65	863	4.8	3.4–6.2	834	13.9	11.6–16.2	1697	9.3	7.9–10.7
Sleep group, h									
≤7	2471	5.4	4.5–6.3	3238	11.0	9.9–12.1	5709	8.6	7.9–9.3
>7	1540	1.3	0.7–1.9	1707	3.7	2.8–4.6	3247	2.6	2.1–3.1
>8	811	1.8	0.9–2.7	759	4.9	3.4–6.4	1570	3.3	2.4–4.2
>9	483	2.9	1.4–4.4	431	9.7	6.9–12.5	914	6.1	4.5–7.7
Annual income, RMB									
≤5000	716	8.5	6.5–10.5	716	16.5	13.8–19.2	1432	12.5	10.8–14.2
5000–20000	2859	3.3	2.6–4.0	3390	8.3	7.4–9.2	6249	6.0	5.4–6.6
>20000	1743	1.7	1.1–2.3	2049	4.9	4.0–5.8	3792	3.4	2.8–4.0
Occupational physical strength									
Light	1562	6.5	5.3–7.7	2601	10.6	9.4–11.8	4163	9.1	8.2–10.0
Moderate	1010	2.1	1.2–3.0	1197	6.5	5.1–7.9	2207	4.5	3.6–5.4
Severe	2746	2.2	1.7–2.7	2357	6.2	5.2–7.2	5103	4.0	3.5–4.5
BMI, kg/m2									
<25	2959	3.8	3.1–4.5	3287	8.3	7.4–9.2	6246	6.2	5.6–6.8
25–30	1962	3.2	2.4–4.0	2268	7.6	6.5–8.7	4230	5.6	4.9–6.3
>30	349	2.3	0.7–3.0	541	8.5	6.1–10.9	890	6.1	4.5–7.7
Marital status									
Married	4949	3.1	2.6–3.6	5657	7.6	6.9–8.3	10606	5.5	5.1–5.9
Single	141	5.0	1.4–8.6	51	17.6	7.1–28.1	192	8.3	4.4–12.2
Widowed	228	9.2	5.4–13.0	447	13.4	10.2–16.6	675	12.0	9.5–14.5
Education level									
≤Primary school	2192	4.2	3.4–5.0	3470	10.2	9.2–11.2	5662	7.9	7.2–8.6
Middle school	2513	3.1	2.4–3.8	2209	5.7	4.7–6.7	4722	4.3	3.7–4.9
>High school	613	2.1	1.0–3.2	476	3.6	1.9–5.3	1089	2.8	1.8–3.8
Chronic diseases									
Yes	1093	8.4	6.8–10.0	1687	14.9	13.2–16.6	2780	12.4	11.2–13.6
No	4225	2.2	1.8–2.6	4468	5.5	4.8–6.2	8693	3.9	3.5–4.3
Vegetables intake (g/week)									
rarely	100	4.0	0.2–7.8	117	14.5	8.1–20.9	217	9.7	5.7–13.6
<1,000 g	396	6.6	4.2–9.0	471	14.2	11.0–17.4	867	10.7	8.6–12.8
1,000–2,000 g	2690	2.6	2.0–3.2	3319	7.4	6.5–8.3	6009	5.3	4.7–5.9
≥2,000 g	2132	3.9	3.1–4.7	2248	7.5	6.4–8.5	4380	5.7	5.0–6.4
Meat intake (g/week)									
rarely	727	9.5	7.4–11.6	1561	13.3	11.6–15.0	2288	12.1	10.8–13.4
<250 g	1200	3.1	2.1–4.1	1791	8.1	6.8–9.4	2991	6.1	5.2–7.0
250–500 g	1607	1.8	1.2–2.5	1641	4.5	3.5–5.5	3248	3.2	2.6–3.8
≥500 g	1784	2.7	1.9–3.5	1162	6.1	4.7–7.5	2946	4.0	3.3–4.7
Fried foods intake (Frequency/week)									
<1 times	1764	5.3	4.3–6.3	2738	11.1	9.9–12.3	4502	8.8	8.0–9.6
2–3 times	2894	2.6	2.0–3.2	2844	5.6	4.8–6.4	5738	4.1	3.6–4.6
≧4 times	660	2.3	1.2–3.4	573	6.1	4.1–8.1	1233	4.1	3.0–5.2
Beans or Bean products intake (Frequency/week)									
rarely	3936	3.7	3.1–4.3	5396	8.4	7.7–9.1	9332	6.4	5.9–6.9
2–3 times	1131	2.7	1.8–3.6	666	5.7	3.9–7.5	1797	3.8	2.9–4.7
≧4 times	251	2.4	0.5–4.3	93	7.5	2.1–12.9	344	3.8	1.8–5.8

### Multivariate Regression Analysis

We divided the sample populations at high risk for depression and the normal population according to the PHQ-9 scores. We conducted a regression analysis on the factors that might influence the depressive symptoms. The results are shown in Table 3. Only gender, amount of sleep time, meat intake and chronic disease were positively correlated with depression symptoms. Annual income, education level, weekly consumption of meat and beans or bean products, and occupational physical activity were negatively associated with depressive symptoms.

**Table 3:** Sex-dependent Risk Factors Associated With Different PHQ-9 Score Classes From the Multivariate Logistic Regression.

Variables	OR	95%CI for OR		P value
		Lower	Upper	
Female	1.762	1.420	2.187	<0.001
Age, yr				
35–45	Reference			
45–55	1.244	0.952	1.625	0.109
55–65	1.055	0.799	0.799	0.708
>65	0.907	0.654	1.257	0.557
annual income				
≤5000	Reference			
5000–20000	0.610	0.492	0.755	<0.001
>20000	0.418	0.319	0.546	<0.001
Sleep time				
7–8	Reference			
≤7	2.967	2.322	3.790	<0.001
8–9	1.290	0.899	1.852	0.166
>9	1.290	0.899	1.852	<0.001
Education level				
≤primary school	Reference			
middle school	0.830	0.684	1.008	0.06
>middle school	0.573	0.385	0.854	0.006
Meat intake				
250–500 g	Reference			
rarely	2.463	1.916	3.166	<0.001
<250 g	1.491	1.150	1.933	0.003
≥500 g	1.494	1.125	1.983	0.005
Beans or bean products intake				
2–3 times	Reference			
rarely	1.783	1.492	2.132	<0.001
≧4 times	1.069	0.774	1.478	0.685
Vegetables intake (g/week)				
1,000–2,000 g	Reference			
rarely	1.263	0.770	2.072	0.355
<1,000 g	1.670	1.284	2.172	<0.001
≥2,000 g	1.221	1.015	1.469	0.034
Occupational physical activity				
Light	Reference			
Moderate	0.572	0.449	0.728	<0.001
Severe	0.602	0.492	0.737	<0.001
Chronic disease				
No	Reference			
Yes	2.532	2.132	3.007	<0.001

No significant difference: Marital status, Smoking, Drinking and Fried food intake.

### Assessment of Life Quality and Depression Symptoms

The subjects were divided into two groups according to the PHQ-9 score. An analysis showed that the physiological, psychological and social relations dimension scores in individuals with a score less than 10 PHQ-9 points were significantly higher compared with individuals with a score greater than 10 for both sexes (*P*<0.001). However, there was no significant difference for the environment dimension. The results are shown in Table 4.

**Table 4:** Comparison between the PHQ-9 score ≥10 group and <10 group in each domain score.

WHOQOL-BREF domain	Male		P	Female		P
	<10 group	≥10 group		<10 group	≥10 group	
Physiological	27.40±3.53	20.10±4.79	<0.001	26.51±3.68	20.10±4.34	<0.001
Psychological	22.55±3.13	16.42±4.31	<0.001	21.88±3.41	16.00±3.82	<0.001
Social relations	11.12±1.53	9.53±1.99	<0.001	11.01±1.51	9.87±1.69	<0.001
Environment	27.34±4.04	23.25±4.27	0.948	27.05±4.14	23.36±4.20	0.669

Score in each domain is represented as the mean±SD.

P<0.05 was considered statistically significant.

## Discussion

To understand the prevalence of depressive symptoms in rural China, we gathered health information from ≥35-year-old adults in rural communities in Northeast China. The data indicated that the prevalence of depressive symptoms in the rural population was 5.9% (Male, 3.4%; Female, 8.1%), which suggests that depression has become a serious health problem in the rural Chinese population. Gender, amount of sleep time and chronic disease, annual income, education level, weekly consumption of meat and beans or bean products, and occupational physical activity were significantly associated with depression symptoms. Systematic epidemiological studies of depression have a history of over half a century, with depression exhibiting an increasing incidence over time. A community-based study found the one-year prevalence of major depressive disorder rose from 3.33% to 7.06% between 1991–92 and 2001–02 in American adults [19]. The National Comorbidity Survey Replication reported that the prevalence of major depressive disorder was 16.2% for the lifetime rate and 6.6% for the 12 month prevalence [20]. The ODIN (Outcome of Depression International Network) study in a European population indicated that the prevalence of depression disorders was 8.56% in 2001 [21]. The survey included 14 European countries and reported that depressive episodes accounted for 4.4% of the total disability adjusted life years (DALYs) in the year 2000, which represented the fourth leading cause of disease burden in women and the seventh leading cause in men [22]. Wurff reported that the prevalence of depression symptoms in the Netherlands was 14.5% [23]. The prevalence of depression symptoms in Australians was 3.2% in 2003, which is far below the level of European countries and the US [24]. Although many studies have been conducted in developed countries, the mental health status of the population in China has been ignored for decades. Chen et al. reported that the prevalence of depression in older individuals was 6.0% in 2005. Pan et al. reported that the prevalence of depressive symptoms in metropolitan regions in middle-aged and elderly individuals in China was 9.5% in 2008 [25]. It appears that the prevalence of depression symptoms is lower compared with the prevalence in the urban population. However, our study suggested that the prevalence of depression symptoms in rural areas is lower compared with Beijing, but is higher compared with Shanghai [26]. Several researchers have also shown that the prevalence of an affective disorder or depression is higher in rural areas compared with urban areas [27], [28]. This increased prevalence may be because individuals in rural areas are more likely to have characteristics that are strongly associated with depression, including poor health status, chronic disease, and poverty. In addition, regional and ethnic differences are also important factors that cannot be ignored. Our study indicated that the prevalence of depression symptoms was higher than some other regions, although it was lower compared with some large cities in China. Nevertheless, with the development of economy and society, the mental health of individuals in rural populations has become a problem of great concern.

The multivariate logistic regression analysis indicated that gender, annual income, amount of sleep per day, weekly consumption of meat and beans or bean products, education level, chronic disease, and occupational physical activity levels were the main risk factors for depression symptoms. Epidemiological investigations of depression abroad showed that marital status, education level and household income are closely related to the prevalence of depression [4]. However, the association between marital status and the prevalence of depression was not found in our study. We also investigated the living habits of a rural population, such as eating habits, smoking and drinking status. Typical Western dietary patterns have been implicated in directly contributing to the incidence of most diseases [29], [30], but a large prospective study found that adherence to a Mediterranean diet was protective against the self-reported development of depression [31]. Bhattacharyya reported that psychological distress is significantly lower with fish intake in both sexes [32]. Colin’s research suggested that a significant decrease in the consumption of the polyunsaturated omega 3 fatty acids, which are present in linseed oil, nuts, soya beans, wheat and cold water fish, are found in the plasma and/or in the membranes of the red blood cells in individuals with major depression [33]. However, dietary patterns are not related to the risk of depression in Japan [34]. This difference may be attributed to the different dietary patterns across countries. Physical activity appears to be a protective factor for depressive symptoms [35] and exerts its effects in a dose-dependent manner [36]. Occupational physical activity accounted for the majority of manual labor in the rural population, and our research shows that heavy labor reduces the risk of depression. Overall, physical activity has unequivocal health benefits for the prevention and treatment of chronic diseases, including depression [37].

Anxiety and depression could decrease immune function and physical activity and increase physical discomfort. The more severe the depression, the worse the health and functional status of the individual. Our study showed that the physical, psychological and social relationship scores of the quality of life in individuals with depressive symptoms (PHQ-9 score≥10) were significantly lower compared with non-depressed individuals (PHQ-9 score<10). These results are consistent with Norberg’s research [38]. However, no significant difference was found in the environment dimension; this may be because the study population was living in a similar environment, and there was not a substantial difference in the degree of economic development between the depressed and non-depressed participants. Individuals who are in a state of depression will suffer from mental and physical agony that affects the quality of life, and family and occupational dysfunction can be risk factors for suicide [39]. Since the rural population accounts for the majority of our population, their physical and mental health needs require our attention.

There are several limitations in our research. First, we did not confirm a definitive diagnosis of depression. Second, we did not perform detailed quantitative analyses of the eating habits of the participants, although this area is an important topic for future research.

In conclusion, the prevalence of depression symptoms is higher than some large cities in China, and psychological situation of rural population needs to pay more attention by our government. Lifestyle changes play an important role in the incidence of mental health, and these changes might be a potential avenue for intervention to mitigate the problem of depression in rural China.
